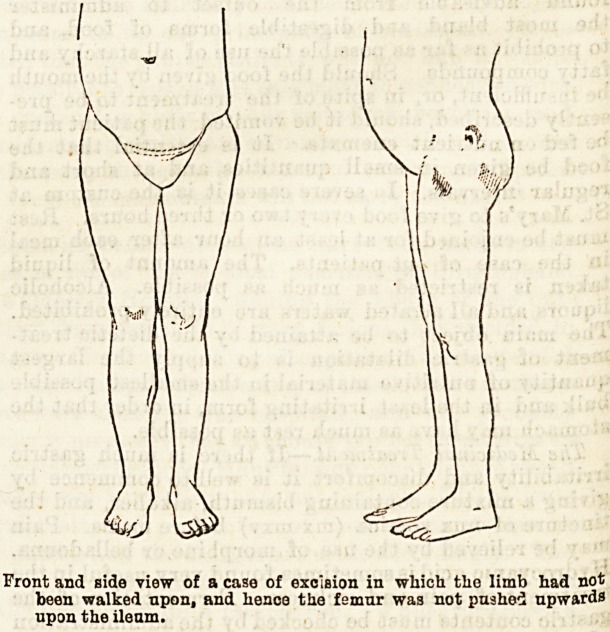# The Treatment of Hip Disease

**Published:** 1893-01-28

**Authors:** G. A. Wright

**Affiliations:** Assistant Surgeon Royal Infirmary, Surgeon Children's Hospital, Manchester, Examiner in Surgery in the University of Oxford


### MANCHESTER AND PENDLEBURY CHILDRENS HOSPITAL.

### The Treatment of Hip Disease.

By G. A. Wright, B.A., M.B. Oxon., F.R.C.S., Assistant Surgeon Royal Infirmary, Surgeon Children's Hospital, Manchester, Examiner in Surgery in the University of Oxford.

Some 150 to 200 cases of hip disease are under treatment during the year at the Children's Hospital, Manchester and Pendlebury; of these the worst cases, such as those in which there are abscesses, or the disease is acute or rapidly progressing, are sent to the hospital, the others are treated as out-patients. For many years our routine method has been the application of a Thomas' splint. After the splint has been fitted on, the child is usually kept lying down for a week or two, and then, if over four years of age, is supplied with a patten and crutches, and allowed to go about. I find that four years is the earliest age at which a child can be trusted to use crutches safely. If all goes on well, the child comes to the out-patient room for inspection every two or three weeks until recovery takes place, the duration of treatment varying according to the stage of the disease and its severity from a few months to two years or more. As soon as all evidence of active disease has passed away, *i.e.*, as soon as all thickening and glandular enlargement, all pain and malposition have disappeared, and mobility has returned to such a degree as is considered to correspond with what can be reasonably expected, considering the extent of the disease in each particular case, the splint is removed, and the child allowed to go about with patten and crutches for a short time each day; the time of freedom is gradually extended until the splint is, after some weeks, left off altogether, and then, after a further period of probation, ranging from weeks to months, the child is allowed to gradually begin walking again on the limb. If at any time there is any evidence of a relapse, the splint is re-applied, and treatment begins anew. If the position of the limb is such that considerable adaptation of the splint would be required, I prefer to send the child to hospital for a short time until the deformity is corrected rather than to correct it by means of the splint itself. A large number of the cases sooner or later require admission to hospital, and this because it is in many cases impossible to get the parents to take sufficient care in keeping on the splint without disturbance, and in preventing the child from falling, or walking, or doing something which disturbs the joint. I believe failure is due to want of attention in carrying out instructions, rather than to defect in the method of treatment, in the majority of those cases which are seen at an early stage.

Should, then, the disease progress, or should an abscess form, the child is admitted to hospital. On admission, if the limb is in bad position a long splint is applied to the sound side, and extension by weights (1lb. per year of age up to six years) to the affected limb until it is straight, or nearly so. Axis traction is not usually employed. If all goes on well, as soon as the disease has become quiescent the child is again sent out in a Thomas' splint. If, however, the symptoms persist, and evidence of suppuration appears, with progressive disease, the joint is opened and usually excised. For excision I employ either the ordinary curved incision behind the trochanter, or, if there is much pelvic disease, a flap operation is performed. I remove the head and neck, with more or less of the shaft, according to the extent of the mischief, clear out the joint cavity, scraping and, if necessary, gouging the acetabulum, thoroughly clean out any abscess cavity, scraping away its walls, then flush out the whole wound with hot perchloride of mercury lotion, inject emulsion of iodoform, suture the wound without drainage, and put the child in a Bryant's double splint. The wound is dressed with iodoform and boric gauze and wood wool wadding. If all goes well the first dressing is about the tenth day, the sutures are then removed, and the child is kept in the splint for three or four weeks, or longer, and then sent out in a Thomas' splint again. Should there be an abscess, without evidence of actively progressive disease, the abscess is laid open and dealt with in similar fashion, without excision of the head of the bone, and sometimes this is done as a preliminary to excision.

Six months or a year are, if possible, allowed to elapse after the wound has healed before the child is allowed to walk upon the limb. This is in order to minimise as much as possible the shortening of the limb resulting from pushing up of the shaft of the bone upon the dorsum ilii.

Front and side view of a case of excision in which the limb had not been walked upon, and hence the femur was not pushed upwards upon the ileum.

Should the wound fail to heal by primary union, a certain time is allowed to elapse (say four or six weeks), and if it remains still unhealed the sinuses are scraped out, and the wound resutured, or allowed to close by granulation.

Amputation is rarely resorted to, and is called for chiefly when, after excision, suppuration persists with extensive femoral or pelvic disease.

Such, briefly, is the method of treatment I usually employ.

During the year 1892 seventeen cases of hip disease have been dealt with by operation; of these ten have been excised. Seven cases of excision healed by primary union, and were discharged after an average treatment of four weeks from the time of operation; one case healed by granulation, and was discharged in two months; and in one instance a wound which had healed by primary union broke down, but healed again, and the child went out in 2½ months.

The last case required scraping again, and was discharged six months after the first operation. One of the cases of primary union was neglected at home, and an abscess formed. It is now nearly well again.

In the second group of seven cases, where abscesses were dealt with without excision of the joint by scraping and suturing, there were three cases in which disease of bone was reached; two of these healed by primary union and one by granulation.

Two other cases had been excised at some previous time; one died from shock after clearing out the abscess, the other recovered in three months.

The other two cases required scraping out a second time, and healed, one in six, the other in three months.

The above cases were under my own care. I have not dealt with those under the charge of my colleague, Mr. Collier.

The results show a great improvement as regards time upon the older methods of treatment, but there is no doubt that unless great care is taken in the subsequent management of the case, there is a distinct danger of recurrence of disease, accompanied by the formation of fresh abscesses.

## Figures and Tables

**Figure f1:**